# Does acute stress influence the Pavlovian-to-instrumental transfer effect? Implications for substance use disorders

**DOI:** 10.1007/s00213-020-05534-8

**Published:** 2020-06-06

**Authors:** Sabine Steins-Loeber, Frank Lörsch, Caroline van der Velde, Astrid Müller, Matthias Brand, Theodora Duka, Oliver T. Wolf

**Affiliations:** 1 Department of Clinical Psychology and Psychotherapy, Otto-Friedrich-University of Bamberg, Markusplatz 3, 96047 Bamberg, Germany; 2 Department of Psychosomatic Medicine and Psychotherapy, Hannover Medical School, Hannover, Germany; 3 Department of General Psychology: Cognition, University of Duisburg-Essen, Duisburg, Germany; 4 Sussex Addiction Research and Intervention Centre, School of Psychology, University of Sussex, Brighton, UK; 5 Department of Cognitive Psychology, Ruhr University Bochum, Bochum, Germany

**Keywords:** Addiction, Nicotine dependence, Socially evaluated cold pressor test

## Abstract

**Rational:**

The ability of conditioned stimuli to affect instrumental responding is a robust finding from animal as well as human research and is assumed as a key factor regarding the development and maintenance of addictive behaviour.

**Objectives:**

While it is well known that stress is an important factor for relapse after treatment, little is known about the impact of stress on conditioned substance-associated stimuli and their influence on instrumental responding.

**Methods:**

We administered in the present study a Pavlovian-to-instrumental transfer (PIT) paradigm with stimuli associated with smoking- and chocolate-related rewards using points in a token economy to light to moderate smokers who also indicated to like eating chocolate. After completion of the first two phases of the PIT paradigm (i.e. Pavlovian training and instrumental trainings), participants were randomly allocated to the socially evaluated cold pressor test or a control condition before the final phase of the PIT paradigm, the transfer phase, was administered.

**Results:**

The presentation of a smoking-related stimulus enhanced instrumental responding for a smoking-related reward (i.e. ‘smoking-PIT’ effect) and presentation of a chocolate-related stimulus for a chocolate-related reward (i.e. ‘chocolate-PIT’ effect) in participants aware of the experimental contingencies as indicated by expectancy ratings. However, acute stress did not change (i.e. neither enhanced nor attenuated) the ‘smoking-PIT’ effect or the ‘chocolate-PIT’ effect, and no overall effect of acute stress on tobacco choice was observed in aware participants.

**Conclusions:**

The established role of stress in addiction appears not to be driven by an augmenting effect on the ability of drug stimuli to promote drug-seeking.

## Introduction

Substance use disorders are a major health problem. While a large number of individuals suffering from a substance use disorder quit substance use without therapeutic interventions (Heyman 2013), there are also individuals who show a chronic course of the disease. Thus for those individuals, high rates of relapse are observed despite the availability of specific pharmacological and psychotherapeutic interventions, and it is important to enhance our understanding of underlying factors. Theories that describe the development and maintenance of substance use disorders stress the important role of both Pavlovian and instrumental learning processes. Thus, it is assumed that stimuli that are regularly associated with the use of a drug become conditioned drug-associated stimuli and are able to elicit conditioned responses and motivate instrumental drug-seeking behaviour (Berridge and Robinson 2016; Everitt and Robbins 2016). Although studies on the association between conditioned responses (e.g. craving) and relapse have provided inconsistent or non-significant results (e.g. Perkins 2009), there are other studies that showed for example that an increase in craving after exposure to smoking-related cues is associated with increased smoking behaviour (Conklin et al. 2015). However, while it is well known that stress is an important factor for the development and maintenance of substance use disorder (Koob 2008; Schwabe et al. 2011), little is known about the potential interplay of stress with learning processes and instrumental behaviour. Previous studies have shown for example that negative mood induction increases tobacco choice (Hogarth et al. 2017; Hogarth et al. 2015a), alcohol choice (Hogarth et al. 2018; Hogarth and Hardy 2018; Hardy and Hogarth 2017) and heroin choice (Hogarth et al. 2019a), and stress increases alcohol choice (Shuai et al. 2020). There are also several studies demonstrating that negative effect or stress increases tobacco motivation, craving and consumption (see review by Heckman et al. 2015). For example, it was found that stress significantly decreases the ability to resist smoking after a brief 3 h (Oberleitner et al. 2018) or an overnight nicotine deprivation (McKee et al. 2011). Some authors propose that stress can disrupt top-down inhibitory control of the dorsolateral prefrontal cortex (Woodcock et al. 2019), promote a transfer to habit behaviour (Schwabe et al. 2011) or enhance stimulus-triggered ‘wanting’ (but not necessarily ‘liking’) of the reward through raising dopamine levels in the nucleus accumbens (Sinha 2001; Hyman et al. 2006; Graf et al. 2013). Another pathway may be that stress increases the expected value of a drug thus driving goal-directed drug choice (Mathew et al. 2017; Shuai et al. 2020; Hogarth et al. 2019a; Hogarth and Hardy 2018; Hogarth et al. 2015a).

Both animal (Peciña et al. 2006 and human studies (Pool et al. 2015, Pritchard et al. 2018, Quail et al. 2017, Pritchard et al. 2018) have found that stress might affect the influence of conditioned stimuli on instrumental responding and attenuate the impact of devaluation procedures (i.e. eating or drinking water to satiety).

The Pavlovian-to-instrumental transfer (PIT) paradigm is an established paradigm to investigate the impact of conditioned stimuli on instrumental responding with the first study using the PIT paradigm to assess drug-related mechanisms dating back to 2007 (Hogarth et al. 2007). The PIT paradigm allows for the assessment of the effects of the Pavlovian conditioned stimuli (Hogarth et al. 2010) on separately trained instrumental reward-related responses. Two different forms of PIT or transfer effects, specific and general transfer, are described which are characterised by different neural substrates. General transfer describes the ability of conditioned stimuli to enhance responding for different rewards, while specific transfer refers to the ability of stimuli to enhance instrumental responding for rewards associated with the same outcome as the stimuli. Hogarth and colleagues used the PIT paradigm in a number of experimental studies and demonstrated for example that the presentation of a tobacco-related stimulus increased performance of a tobacco-related response (Hogarth et al. 2015b; Hogarth and Chase 2012; Hogarth 2012; Hogarth and Chase 2011). Similarly, Martinovic and colleagues (Martinovic et al. 2014) demonstrated an ‘alcohol PIT’ effect in social drinkers, as participants increased responding by pressing a key associated with the award of ‘beer-points’ in the presence of a beer-related stimulus. Nevertheless, although severity of dependence increases substance-related instrumental responding (see Hardy et al. 2018, Hogarth 2020 for a review), there are a number of studies that demonstrated that dependence severity is not associated with the PIT effect (see Hardy et al. 2017 for a review). For example, with regard to nicotine dependence, Hogarth and colleagues found in four independent studies no association between severity of nicotine dependence and a ‘smoking PIT’ effect (Hogarth et al. 2015b; Hogarth and Chase 2011, 2012; Hogarth 2012). In addition, Hogarth and colleagues (Hogarth et al. 2019b) reported no differences between substance-dependent individuals and healthy controls with regard to the PIT effect in response to natural rewards. In contrast, Garbusow and colleagues (Garbusow et al. 2014) investigated PIT effects in patients suffering from alcohol use disorder and found that patients compared with controls more frequently showed a PIT effect. Using functional imaging, it was also found that PIT-related neural activation was a valid predictor for relapse (Garbusow et al. 2016; Sekutowicz et al. 2019). However, the paradigm developed by this research group to assess the PIT effect does not resemble any other PIT paradigm used in animal as well as human research so far as alcohol-related cues were presented as distractors in the background. Thus, it is not clear whether this paradigm measures the same mechanisms as a standard PIT paradigm calling the interpretation of the results into question, especially as standard tobacco-related and alcohol-related PIT paradigm measures do not correlate with dependence as outlined above. Interestingly, the psychological mechanisms underlying the PIT effect remain a matter of debate, and only recent research using outcome devaluation procedures demonstrated that specific PIT effects are driven by propositional beliefs about the role of the stimuli in signalling the response outcome relationships and do not necessarily reflect habitual behaviour (Seabrooke et al. 2017; Seabrooke et al. 2019). This observation is in line with the finding that PIT effects are only observed in study participants aware of the stimulus-response-outcome contingencies and can be abolished by instructions that contradict the explicit outcome expectancy (e.g. Seabrooke et al. 2016; Hogarth et al. 2014). Few studies have investigated the effects of experimentally manipulated acute stress on transfer effects in rodents and, to the best of our knowledge, there are only two human studies so far (Pool et al. 2015; Pritchard et al. 2018). In rats, Peciña and colleagues (Peciña et al. 2006) found that a dose of 500 mg corticotropine-releasing factor injected into the medial shell of the nucleus accumbens selectively enhanced the ability of a conditioned reward-related stimulus to increase instrumental responding in a single lever paradigm. Contrary to these findings, Pielock and colleagues (Pielock et al. 2013) found that acute stressors did not affect the PIT effect. In 2015, Pool and colleagues (Pool et al. 2015) were the first to investigate the effects of acute stress on the transfer effect in humans using a single lever paradigm. Participants thus learned during instrumental training to press a handgrip to trigger the release of a rewarding chocolate odour. During Pavlovian training, they learned to associate an abstract symbol with the chocolate odour (CS+) and another symbol with odourless air (CS−). After administration of the socially evaluated cold pressor test (SECPT) or a non-stress control induction, participants completed the transfer phase. It was found that stress increased responding for the chocolate odour after presentation of the CS+ (i.e. PIT effect) in the stress, but not the non-stress condition without affecting liking of the odour. Only recently, Pritchard and colleagues (Pritchard et al. 2018) used a PIT paradigm with natural rewards (i.e. mineral water, chips) and found that acute stress did not affect the PIT effect, but attenuated the impact of a devaluation procedure (i.e. drinking water until satiety) on instrumental choice suggesting that stress impaired the retrieval of the expected value of the outcome. However, as all of these studies used natural rewards, it is not clear whether acute stress enhances the impact of conditioned stimuli on instrumental responding for drug-related rewards. This would be important to enhance our understanding of acute stress effects with regard to the maintenance of addictive behaviours.

Against this background, the aim of the present study was to investigate the influence of acute stress on the impact of conditioned stimuli related to drug or natural rewards on instrumental responding for these rewards. We administered a PIT paradigm with stimuli related to smoking and chocolate rewards. Given the literature on possible impairing effects of stress on learning (Schwabe and Wolf 2010; Vogel et al. 2018), participants were exposed to the SECPT or a control condition after they underwent the Pavlovian and instrumental training phase but before the transfer phase. We hypothesised that presentation of the smoking-related stimulus would be associated with an increase in instrumental responding for the smoking-related reward (i.e. ‘smoking PIT’ effect), and that presentation of the chocolate-related stimulus would be associated with an increase in instrumental responding for the chocolate-related reward (i.e. ‘chocolate PIT’ effect). We expected that acute stress would be associated with a general increase in instrumental responding for the smoking-related reward and enhance the impact of the smoking-related stimulus on instrumental responding for the smoking-related reward compared with the no cue condition (i.e. ‘smoking PIT’ effect). While we assumed that stress would also enhance the ‘chocolate PIT’ effect, we expected a less pronounced effect compared with the ‘smoking PIT’ effect. As previous studies demonstrated that the severity of nicotine dependence is not associated with the PIT effect, the expected finding that stress increases the ‘smoking PIT’ effect would also underline the validity of the PIT paradigm as a marker for dependence.

## Materials and methods

### Participants

Fifty-nine male and female participants aged between 18 and 35 were recruited from the university student and general population of Bamberg, Germany, via posters and social media platforms. Inclusion criteria were self-reported light to moderate smoking and liking of chocolate. Exclusion criteria for females were pregnancy or breastfeeding and intake of oral contraceptive to avoid confounding effects regarding cortisol responses (Schwabe and Wolf 2009). For female participants, testing was scheduled within the last two weeks of their menstrual cycle. Participants were instructed to abstain from the use of alcohol for at least 24 h, not to consume coffee or tea or to exercise for at least six hours, to refrain from smoking for three hours and not to eat for at least one hour before the test session. The study adhered to the Declaration of Helsinki and was approved by the ethics committee of the University of Bamberg. All participants provided written informed consent. Participants were compensated for their time financially with 15€ or received course credits.

### General procedure

**Fig. 1:**
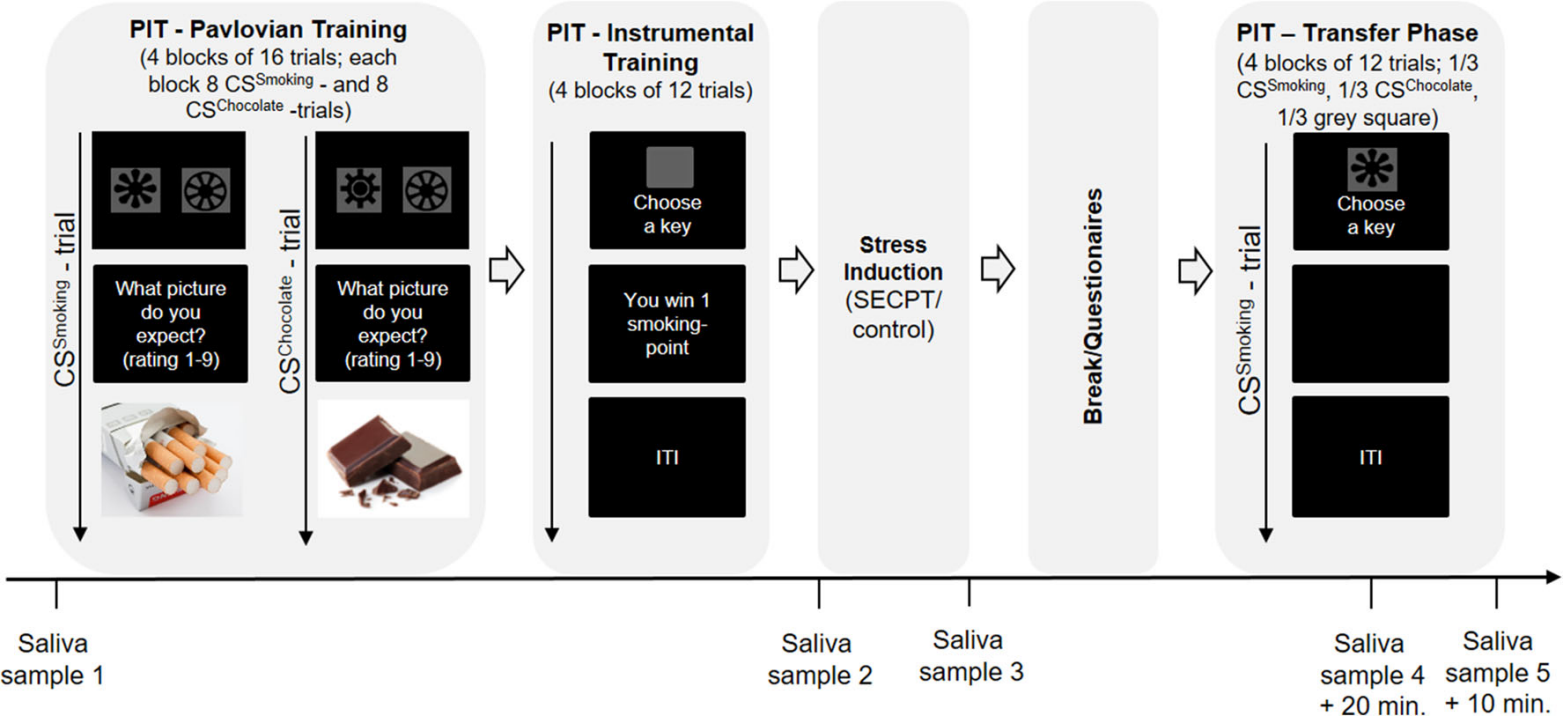
Study procedure; SECPT socially evaluated cold pressor test

Testing comprised a single test session (see Fig. 1) that lasted about 80 min and was scheduled between 1230 and 1700 h to control for diurnal cycle of cortisol (Dickerson and Kemeny 2004). On arrival at the laboratory, participants completed different questionnaire measures. Their subjective stress level was assessed, and a first saliva sample (T1) for the measurement of cortisol was collected to familiarise participants with the procedure. Then, the first two phases of the PIT paradigm, i.e. Pavlovian training and instrumental training, were administered. Upon completion, participants were randomly exposed to the SECPT or a control condition. Then, further questionnaires that were not scored were administered to allow cortisol responses to increase before the final phase of the PIT paradigm, the transfer phase, was administered. Further assessments of the subjective stress level and saliva samples were collected as outlined below.

### Socially evaluated cold pressor test

The SECPT was used as a valid procedure to induce subjective stress and to activate the sympathetic nervous systems and the hypothalamus-pituitary-adrenal (HPA) axis (Schwabe et al. 2008). As previously described (Schwabe and Wolf 2009; Schwabe et al. 2008), participants were asked to immerse their right hand up to and including the wrist for three minutes (or until they could no longer tolerate it) into ice water (0–2 °C). During hand immersion, they were videotaped, and they were informed that facial expressions would be analysed.

In the control condition, participants immersed their right hand up to and including the wrist for three minutes into lukewarm water (35–37 °C); they were not videotaped.

Saliva samples were collected before Pavlovian training (T1), immediately after completion of instrumental training (i.e. before the stress procedure, T2), immediately after the stress or control procedure (T3), after the first two blocks of the transfer phase (T4) and immediately after the end of the transfer phase (T5) (see Fig. 1 for a timeline). At the same time points, participants were asked to rate how stressed they felt on a visual analogue scale ranging from 0 (= not at all) to 100 (= very much). For saliva sampling, Salivettes (Sarstedt, Nürmbrecht, Germany) were used and samples were kept at − 18 °C until analysis. Cortisol concentrations were determined in duplicates using a cortisol enzyme-linked immunosorbent assay (Demeditec, Kiel, Germany). Inter- and intra-assay coefficients of variance were below 8%.

### Pavlovian-to-instrumental transfer paradigm

The PIT paradigm was identical to the procedure previously used in our laboratory (Vogel et al. 2018) with the exception that experimental stimuli related to smoking or chocolate were used. Thus, we describe the task here only in short.

During *Pavlovian training*, participants had to learn that one of four stimuli (CS^S^) predicted the presentation of smoking-related pictures, while another stimulus (CS^C^) predicted chocolate-related pictures; the other two stimuli (X, Y) served as control stimuli. Thus in each trial, one of four possible stimulus pairs appeared and the question was presented: ‘Do you think you will see a smoking- or a chocolate-related picture? 1 = chocolate -picture, 5 = I don’t know, 9 = smoking –picture’. Then either a smoking- or a chocolate-related picture was presented. The pictures were chosen randomly from a set of 32 smoking- and 32 chocolate-related pictures matched with regard to valence and arousal based on participant ratings in an independent pilot study with no stress induction. Participants completed four blocks of sixteen trials (64 trials in total) with each block containing eight CS^S^ and eight CS^C^ trials.

An *emotional evaluation* of the different stimuli was administered before and after Pavlovian training. Each stimulus was presented twice, in random order, and participants answered the questions: ‘How pleasant do you find this picture on a scale from 1 - 9? (1 = not pleasant at all, 9 = very pleasant)?’ and ‘How arousing do you find this picture on a scale from 1 - 9? (1 = not arousing at all, 9 = very arousing)?’

In *instrumental training*, two different instrumental responses (i.e. button presses) were established to achieve either smoking-related (i.e. red coins with the symbol of a cigarette) or chocolate-related rewards (i.e. purple coins with a chocolate bar). Participants were instructed that they have the possibility to win either smoking- or chocolate-related points by pressing one of two different response keys repeatedly. In each trial, one of the responses was selected at random to be reinforced with a 50% contingency for each response in each block. Instrumental training consisted of four blocks of 12 trials, and after each block, participants were asked to transfer the points they had achieved in two initially empty boxes labelled ‘Your smoking points’ and ‘Your chocolate points’.

The *transfer phase* started as a continuation of instrumental training, and participants were informed that they will now sometimes see some stimuli, but instructions did not imply that the pictures were informative to which response key was reinforced. In 1/3 of the trials, a grey square appeared as control stimuli, while in another 1/3, the grey square was replaced with the CS^S^ and in the final 1/3 with the CS^C^. There were four blocks of 12 trials, and participants did receive feedback only about their total winnings at the end of the transfer phase to preclude new learning.

The experimental procedure was programmed with Presentation® software (Version 19.0, www.neurobs.com).

### Questionnaires

The *Fagerstroem test of Nicotine Dependence* (*FTND*) (Heatherton et al. 1991) is a six-item questionnaire to assess nicotine consumption and severity of nicotine dependence. A maximum score of 10 can be achieved. In the present sample, Cronbach’s α equalled 0.55.

The chocolate version of the *Food Cravings-questionnaire-trait reduced* (*FCQ-T-r*) (Meule and Hormes 2015) was administered to provide a subjective measure of the severity of chocolate craving and loss of control over chocolate consumption. The FCQ-T-r comprises 15 items that are scored from 1 to 6. In the present sample, Cronbach’s α was 0.93.

### Data analyses

Mean expectancy ratings during Pavlovian training were analysed using repeated measures analysis of variance with stimulus (smoking, chocolate) and block (1, …, 4) as repeated measure factors and stress condition (SECPT, control) as group factor. Awareness of the experimental contingencies was calculated as previously suggested (Hogarth et al. 2006) by coding participants as aware if they expected in the final block of Pavlovian training in CS^S^ trials the smoking-related pictures with a significantly higher probability than in CS^C^ trials. Emotional ratings of the CS^S^, CS^C^ and combined control stimuli (X/Y) were entered into an ANOVA with time (before, after Pavlovian training) and stimuli (CS^S^/CS^C^, X/Y) as the repeated measures factors.

Instrumental responding during instrumental training and in the transfer phase was analysed by assessing the percentage of response choice of the smoking-related compared with the chocolate-related key. In addition, we calculated the response rate (in Hz) by averaging the total number of presses on the smoking-related or chocolate-related key in each trial and divided the resulting score by the duration of the response window (i.e. 2 s), and the number of trials in which the smoking- or chocolate-related key was chosen. For the transfer phase, response choice as well as response rate were calculated separately for trials in which the CS^S^, CS^C^ or the grey square was presented. Then, differences with regard to response choice and response rate were analysed using repeated measures analyses of variance. Stress condition (SECPT, control) as well as awareness of the experimental contingencies were entered as group factors. Based on previous studies (Paul et al. 2018), the analyses were rerun excluding cortisol non-responders (*n* = 14), i.e. participants who showed an increase in cortisol of less than 1.5 nmol/L (Miller et al. 2013).

All analyses were performed using IBM SPSS Statistics (Version 25). The assumptions of all statistical procedures applied were checked. In the case of violation of the assumption of homogeneity of variances, Greenhouse-Geiser-adjusted degrees of freedom are reported. If appropriate, partial eta^2^ (*ɳp*^*2*^) as measure of effect size is reported. A significance level of α < 0.05 was considered as significant. For significant main effects, post hoc analyses with Bonferroni-corrected *t* tests were used.

## Results

### Sample characteristics

Participants (*n* = 59, 53% females) in the SECPT and the control condition did not differ significantly with regard to age (*t*(57) = −0.33, *P* = .74), gender (*Χ*^2^(1) = 0.02, *P* = .88), severity of nicotine dependence (*t*(57) = 0.58, *P* = .56) and chocolate craving (*t*(57) = 0.02, *P* = .99). See Table 1 for descriptive data.

**Table 1:** Sample characteristics of participants exposed to the socially evaluated cold pressor test (SECPT) or the control condition

Variable	Stress condition	
	SECPT (*n* = 28)	Control (*n* = 31)
Gender		
Female (*n* (%))	15 (54)	16 (52)
Male (*n* (%))	13 (46)	15 (48)
Age (years) (mean/(SD))	24.11 (2.83)	23.84 (3.34)
FTND (mean/(SD))	1.46 (1.62)	1.71 (1.62)
FCQ-T-r (mean/(SD))	37.43 (12.89)	37.48 (14.00)

*FTND*, Fagerstroem Test of Nicotine Dependence; *FCQ-r*, Food Cravings-Questionnaire-trait reduced

### Subjective and physiological responses to the stress induction

Subjective ratings of stress as well as salivary cortisol responses verified the success of the stress induction. Regarding subjective ratings (see Table 2 for descriptive data), we found a significant main effect of time (*F*(3.02,165.80) = 6.29, *P* < .001, *ɳp*^*2*^ = 0.10), which was qualified by a significant time by condition interaction effect (*F*(4,220) = 7.62, *P* < .001, *ɳp*^*2*^ = 0.12). Post hoc tests confirmed that participants in the stress condition reported significantly more stress directly after the stress induction (T3) (*t*(57) = − 3.29, *P*_corr_ = .01), while the groups did not differ at T1, T2, T4 and T5 (all *t*s *≤* 1.28, all *P*s *≥* = .21). No significant gender differences were observed (all *F*s *≤* 1.69, all *P*s *≥* = .20).

**Table 2:** Subjective stress ratings and cortisol responses (mean, SD) before and after the socially evaluated cold pressor test (SECPT) or the control condition

Variable/time	Stress condition	
	SECPT (*n* = 28)	Control (*n* = 31)
Subjective stress rating		
T1	20.46 (20.64)	27.35 (20.78)
T2	24.07 (25.33)	25.13 (16.77)
T3	38.25 (24.32)	19.65 (19.06)*
T4	19.82 (22.92)	18.87 (18.59)
T5	16.96 (20.02)	18.52 (19.18)
Cortisol response (nmol/L)		
T1	13.58 (9.31)	14.14 (9.40)
T2	10.54 (6.03)	10.47 (5.66)
T3	9.82 (5.44)	9.52 (5.06)
T4	12.60 (6.06)	8.34 (4.07)*
T5	12.01 (6.61)	7.65 (3.60)*

T1 baseline, T2 after instrumental training/before the SECPT, T3 1 min after the SECPT, T4 20–30 min after the SECPT, T5 after the transfer phase

*P_corr_ < 0.05

**Fig. 2:**
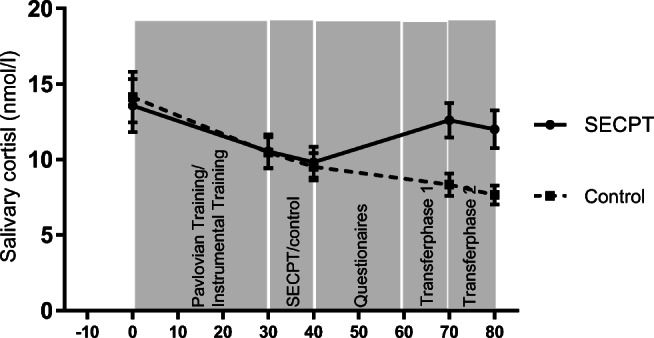
Salivary cortisol responses (in nanomoles per litre) of participants exposed to the socially evaluated cold pressor test (SECPT) and the control condition (mean and SEM)

Regarding cortisol responses, a significant main effect of time (*F*(1.36,74.98) = 14.96, *P* < .001, *ɳp*^*2*^ = 0.21) which was qualified by a significant time by condition interaction effect (*F*(4,220) = 7.15, *P* < .001, *ɳp*^*2*^ = 0.12) emerged. Post hoc tests confirmed that participants in the stress condition had significantly higher salivary cortisol levels at T4 (*t*(46.51) = − 3.14, *P*_corr_ = .01) and T5 (*t*(40.76) = − 3.10, *P*_corr_ = .01), while the groups did not differ from T1 to T3 (all *t*s *≤* 0.23, all *P*s *≥* = .82) (see Fig. 2 for an illustration). A significant main effect of gender (*F*(1,55) = 5.56, *P* = .02, *ɳp*^*2*^ = 0.09) indicated that male participants had higher cortisol levels than female participants at all measurements (male participants, mean = 12.56, SD = 6.23; female participants, mean = 9.26, SD = 4.51). However, gender did not affect the increase in cortisol as indicated by non-significant gender-related interaction effects (all *Fs ≤* 1.47, all *Ps ≥* = .21) suggesting that the stress induction was successful in male as well as female participants.

### Pavlovian training

#### Expectancy ratings and awareness of the experimental contingencies

**Fig. 3:**
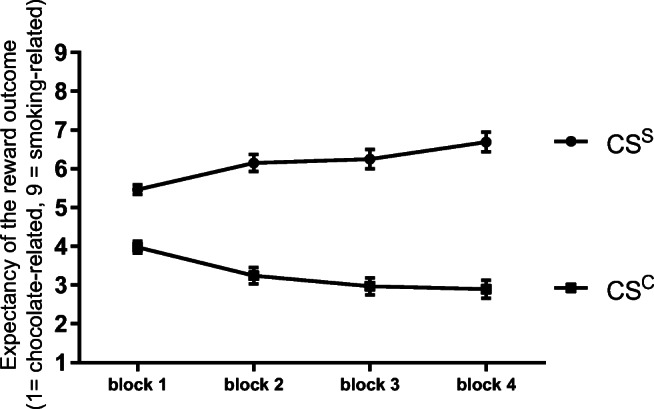
Discrimination between a stimulus which predicted a smoking-related (CS^S^) and which predicted a chocolate-related (CS^C^) reward outcome increased over time (mean and SEM)

*Expectancy ratings* indicated that participants learned over time to discriminate between the stimulus predicting a smoking-related picture and the stimulus predicting a chocolate-related picture (see Fig. 3). There was a main effect of stimulus (*F*(1,57) = 78.28, *P* < .001, *ɳp*^*2*^ = 0.58) which was qualified by a significant stimulus by block interaction effect (*F*(2.15,122.77) = 15.84, *P* < .001, *ɳp*^*2*^ = 0.22). Post hoc tests indicated that the expectancy of smoking-related pictures was significantly higher in CS^S^ than in CS^C^ trials in all four blocks of Pavlovian training (all *t*s ≥ 6.45, all *P*s ≤ .001). All other effects, especially any main or interaction effects including the later allocation to the stress or control condition, were not significant (all *F*s *≤* 0.95, all *P*s *≥* .42).

Fifty-four percent of the participants were classified as *aware* of the experimental contingencies as indicated by significantly higher expectancy of smoking-related pictures in CS^S^ than in CS^C^ trials in the final block of Pavlovian training. Aware and unaware participants did not differ significantly with regard to age (*t*(57) = − 1.29, *P* = .20), gender (*Χ*^*2*^(1) = 0.01, *P* = .92), severity of nicotine dependence (*t*(57) = − 1.82, *P* = .07) or chocolate craving (*t*(57) = 0.69, *P* = .49). There was also no significant difference between the percentage of aware and unaware participants allocated to the stress or the control condition (*Χ*^*2*^(1) = 0.18, *P* = .67).

#### Emotional ratings of the experimental stimuli

Pavlovian training did not affect the *pleasantness ratings* for the different experimental stimuli; we found neither a significant main effect of stimulus (*F*(2,58) = 1.35, *P* = .26, *ɳp*^*2*^ = 0.02) or time (*F*(1,58) = 0.45, *P* = .51, *ɳp*^*2*^ = 0.01) nor a significant stimulus by time interaction (*F*(2,58) = 2.02, *P* = .14, *ɳp*^*2*^ = 0.03). Similar results were observed when this analysis was rerun with aware participants only.

With regard to *arousal ratings*, we found a significant main effect of time (*F*(1,58) = 22.07, *P <* .001, *ɳp*^*2*^ = 0.28), while the main effect of stimulus (*F*(1.81,104.67) = 1.58, *P* = .21, *ɳp*^*2*^ = 0.03) as well as the stimulus by time interaction (*F*(2,116) = 0.23, *P* = .79, *ɳp*^*2*^ = 0.00) were not significant. Post hoc tests indicated that arousal ratings for all experimental stimuli increased during the Pavlovian training (all *t*s ≥ − |2,46|, all *P*s *≤* .0.02). While subsequent analysis with aware participants only revealed a significant main effect of stimulus (*F*(2,62) = 3.74, *P* = .03, *ɳp*^*2*^ = 0.11), post hoc analysis indicated that this effect was not reliable (all *Ps* ≥ .06).

### Instrumental training

During instrumental training, participants responded overall in 96.12% of the trials. Participants choose significantly more often to press the smoking-related key (57.29% of the trials) compared with the chocolate-related key (42.71% of the trials; *F*(1,57) = 6.99 *P* = .01, *ɳp*^*2*^ = 0.11). No significant group differences were observed between participants who were in the next step allocated to the stress or control condition (stress condition by response choice interaction, *F*(1,57) = 0.82, *P* = .37, *ɳp*^*2*^ = 0.01).

Similar results were observed with regard to response rate, as participants pressed faster on the smoking-related compared with the chocolate-related key (*F*(1,57) = 6.34 *P* = .02, *ɳp*^*2*^ = 0.10). Again, no significant group differences with regard to the stress condition emerged (*F*(1,57) = 1.64 *P* = .21, *ɳp*^*2*^ = 0.03).

Correlation analysis indicated that the severity of nicotine dependence was significantly positively correlated to response choice of the smoking-related key (*r* = 0.37, *P* = .004), while no association between chocolate craving and response choice or rate of the chocolate-related key was found.

### Transfer

Participants responded in 95.97% of the trials in the transfer phase. A significant main effect of stimulus (*F*(2,110) = 36.22, *P* < .001, *ɳp*^*2*^ = 0.40) was found which was qualified by a significant stimulus by awareness interaction (*F*(2,110) = 35.99, *P* < .001, *ɳp*^*2*^ = 0.40). The interaction stimulus by awareness by stress condition effect was not significant (*F*(2,110) = 0.10, *P* = .91, *ɳp*^*2*^ = 0.00) as was the stress by awareness interaction effect (*F*(1,55) = 0.63, *P* = .43, *ɳp*^*2*^ = 0.01).

**Fig. 4:**
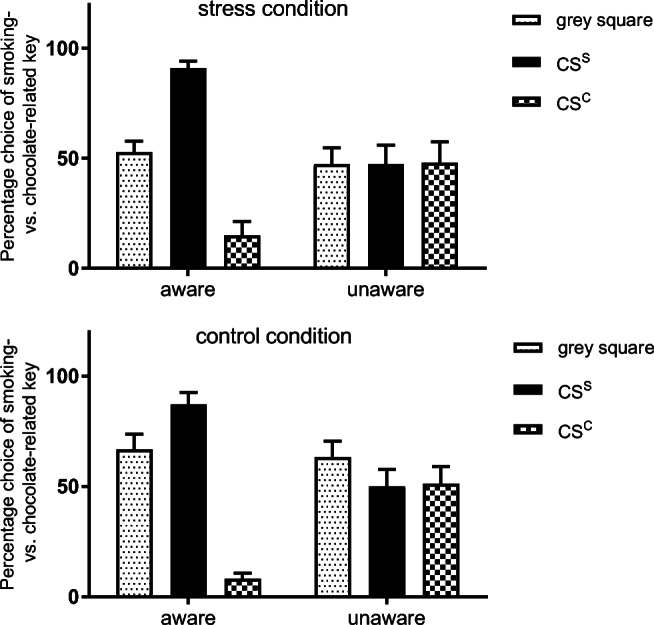
In the stress (upper panel) as well as the control condition (lower panel), aware participants showed a ‘smoking PIT’ effect as well as a ‘chocolate PIT’ effect as indicated by percentage choice of the key associated with the smoking-related reward outcome after presentation of the grey square, the smoking-related stimulus (CS^S^),and the chocolate-related stimulus (CS^C^) (mean and SEM). See text for further details

Post hoc tests indicated a ‘smoking PIT’-effect as well as a ‘chocolate PIT’ effect for aware participants in the stress as well as the control condition. Thus, aware participants choose more often to press the smoking-related key when the CS^S^ was presented compared with presentation of the grey square (stress condition, *t*(15) = 5.59, *P*_corr_ < .001; control condition, *t*(15) = 3.15, *P*_corr_ = .02) and the CS^C^ (stress condition, *t*(15) = 8.37, *P*_corr_ < .001; control condition, *t*(15) = 11.37, *P*_corr_ < .001) (‘smoking PIT’ effect). In line with this, response choice of the smoking-related key was significantly lower when the CS^C^ was presented compared with the grey square (stress condition, *t*(15) = − 5.88, *P*_corr_ < .001; control condition, *t*(15) = − 7.72, *P*_corr_ < .001) indicating that presentation of the CS^C^ increased responding on the chocolate-related key (‘chocolate PIT’ effect). For unaware participants, no significant differences emerged (all *t*s ≤ |− 1.80|, all *P*s ≥ .09). See Fig. 4 for an illustration of the results.

A post hoc analysis excluding cortisol non-responders confirmed the reported results as the main effect of stimulus (*F*(2,84) = 29.73, *P* < .001, *ɳp*^*2*^ = 0.42), and the awareness by stimulus interaction effect (*F*(2,84) = 23.80, *P* < .001, *ɳp*^*2*^ = 0.36) remained significant, while the stimulus by awareness by stress condition effect again was not significant (*F*(2,84) = 0.14, *P* = .87, *ɳp*^*2*^ = 0.00).

Regarding *response rate*, a significant main effect of stimulus (*F*(2,110) = 6.41, *P* < .01, *ɳp*^*2*^ = 0.10) which was qualified by a significant stimulus by awareness interaction (*F*(2,110) = 6.87, *P* < .01, *ɳp*^*2*^ = 0.11) indicated that aware participants pressed the smoking-related key with a higher frequency when the CS^S^ was presented compared with presentation of the CS^C^ (*t*(31) = 3.76, *P*_corr_ < 01). However, responding did not differ from presentation of the grey square (*t*(31) = 1.80, *P =* .08)*.* All other effects did not achieve significance (all *F*s *≤* 1.26, all *P*s *≥* .29). No changes with regard to the effects of the stress induction were observed when only cortisol responders were included in the analysis.

Correlation analysis (see Table 3 for details) indicated that in instrumental training, the severity of nicotine dependence was positively associated with instrumental responding for the smoking-related reward and, in the transfer phase, increased overall responding for the smoking-related reward as well as after presentation of the grey square and the CS^S^. In contrast, the severity of nicotine dependence was not associated with the magnitude of the ‘smoking PIT’ effect. No significant associations between the severity of chocolate craving and chocolate-related responding were observed, although the ‘chocolate PIT’ effect was positively associated with severity of nicotine dependence.

**Table 3:** Correlation matrix showing associations between severity of use patterns and reward-related instrumental responding

	(1)	(2)	(3)	(4)	(5)	(6)	(7)	(8)
(1) FTND								
(2) FCQ-r	0.06							
(3) IT choice of smoking-related key (%)	0.37*	− 0.21						
(4) TP overall choice of smoking-related key (%)	0.32*	− 0.05	0.56*					
(5) TP choice of smoking-related key after square (%)	0.34*	− 0.06	0.56*	0.62*				
(6) TP choice of smoking-related key after CS^S^ (%)	0.34*	0.07	0.31*	0.38*	0.03			
(7) TP choice of smoking-related key after CS^C^ (%)	− 0.15	− 0.05	− 0.04	0.41*	0.07	− 0.45*		
(8) magnitude ‘smoking-PIT’-effect	0.04	0.09	− 0.12	− 0.11	− 0.63*	0.76*	− 0.39*	
(9) magnitude ‘chocolate-PIT’-effect	0.34*	0.00	0.40*	0.08	0.60*	0.38*	− 0.76*	− 0.10

*FTND*, Fagerstroem Test of Nicotine Dependence; *FCQ-r*, Food Cravings-Questionnaire-trait reduced; *IT*, instrumental training; *TP*, transfer phase

*P < .05

## Discussion

Our results first of all replicated previous research findings (Vogel et al. 2018; Hogarth et al. 2019b; Hogarth and Chase 2011, 2012; Hardy et al. 2017; Hardy et al. 2018) indicating that appetitive reward-related stimuli affect instrumental responding for these rewards (i.e. specific PIT effect). Thus, in the transfer phase, the presentation of the stimulus related to smoking (CS^S^) increased responding for a smoking-related reward (i.e. ‘smoking PIT’ effect), while the presentation of a stimulus related to chocolate (CS^C^) increased responding for a chocolate-related reward (i.e. ‘chocolate PIT’ effect). This effect was only observed in participants who were aware of the experimental contingencies which are in line with numerous previous studies that found that knowledge of the experimental contingencies is necessary for the PIT effect (Hogarth et al. 2014; Seabrooke et al. 2016).

Regarding acute stress effects on the impact of conditioned stimuli on instrumental responding, we found no significant differences between stressed and non-stressed participants, although results from subjective ratings as well as cortisol responses indicated that our stress indication procedure was effective. Thus, stress did not increase overall tobacco choice and did not affect instrumental behaviour following presentation of the conditioned stimuli as indicated by non-significant awareness by stress interaction and stimulus by awareness by stress interaction effects. Backing up this result by restricting the analysis on stress effects on the impact of conditioned stimuli on instrumental responding to cortisol responders only supported this finding, as the interaction effect was not significant. Thus, our main hypotheses that acute stress would further promote the impact of the presentation of the smoking-related stimulus as well as the chocolate-related stimulus on instrumental responding for these rewards with greater stress-related effects for the smoking-related compared with the chocolate-related reward were not supported.

Several studies report that stress increases tobacco motivation (see Heckman et al. 2015 for a review). Thus, the fact that in the present study that stress was not associated with an increase in overall tobacco choice is somewhat unexpected. Although subjective and cortisol responses indicated that the stress induction was successful, the SECPT might not be a motivator of tobacco seeking. Interestingly, using a pain induction procedure with heat, Moskal and colleagues (Moskal et al. 2018) found that pain increased alcohol motivation; in contrast, Brady and colleagues (Brady et al. 2006) used the cold pressor test and found that 26.5% of the alcohol-dependent patients showed an increase in craving in response to the stress induction. Thus, different effects might be observed depending on the stress induction procedure used.

The present finding that acute stress did enhance neither the ‘smoking PIT’ nor the ‘chocolate PIT’ effect is in line with findings by Pritchard and colleagues (Pritchard et al. 2018), who investigated whether negative emotional appraisal affects retrieval of outcome values. In this study, negative emotional stimuli were used to influence the emotional state of participants. Thus compared with a control condition with neutral pictures, participants in the negative emotional appraisal condition reported significantly stronger feelings of anxiety, depression, anger, fatigue and confusion. Similarly as in the present study, participants in both conditions showed a PIT effect, and no significant group differences emerged. Although Pritchard and colleagues (Pritchard et al. 2018) investigated negative emotional appraisal, and not stress effects, the results from this study and the present one support the assumption that negative mood and feelings of stress do not affect the impact of conditioned stimuli on reward-related instrumental responding. However, Pritchard and colleagues (Pritchard et al. 2018) also devalued one of the rewards (i.e. instructed participants to drink water until satiety), and this experimental manipulation did only reduce instrumental responding for the reward in the control condition, while participants in the negative emotional appraisal condition still responded for the devalued outcome. As previously outlined by Hogarth and colleagues (Hogarth et al. 2019b; Seabrooke et al. 2017; Seabrooke et al. 2019), the PIT paradigm assesses goal-directed rather than habitual behaviour as indicated by different experimental studies demonstrating that response choice in a PIT task is influenced by reward value and expected outcome probability indicating goal-directed rather than habitual behaviour (Seabrooke et al. 2017). In line with this, Pritchard and colleagues (Pritchard et al. 2018) interpret their finding as evidence that in a negative emotional state, the capacity to retrieve the expected value of instrumental outcomes and thus goal-directed behaviour can be impaired. As we did not implement in the present study an outcome devaluation procedure, our conclusions are limited to the finding that acute stress does not seem to affect the PIT effect for drug-related as well as natural rewards. The result that stress does not affect the PIT effect for natural rewards is thereby a failure to replicate the results from Pool and colleagues (Pool et al. 2015), as in this study acute stress did enhance responding for a chocolate odour. However, the present study differs from the study by Pool and colleagues (Pool et al. 2015) in several aspects, for example the administration of a choice paradigm and the use of chocolate coins as reward in the present study compared with a single lever paradigm or the use of chocolate odour by Pool and colleagues (Pool et al. 2015), which might explain diverging findings. For future studies, it would be interesting to implement an outcome devaluation procedure in a PIT paradigm as described in the present study to enhance our understanding of the impact of acute stress effects on instrumental responding for drug-related as well as natural rewards and mechanisms underlying the maintenance of reward-related behaviour.

There are a few limitations that should be acknowledged when interpreting the present findings. Firstly, only light to moderate smokers were included to avoid confounding effects of nicotine withdrawal after abstaining for at least three hours prior to the test session from nicotine. We cannot exclude that different findings would have been observed with more severe dependent participants given that previous research demonstrated a positive association between severity of dependence and preferential choice of the drug (Hardy et al. 2018; Hogarth and Chase 2011, 2012; Hogarth et al. 2019b). However, these studies also demonstrated that the severity of nicotine dependence was not associated with the magnitude of the PIT effect, which was also observed in the present study. Thus, although for example studies on cue reactivity in substance dependence suggested a complex association between severity of dependence and cue reactivity (Smolka et al. 2006; Vollstädt Klein et al. 2011), the PIT paradigm might be a poor assay of addiction as it seems not to be affected by dependence severity and stress. Importantly, this observation might be due to ceiling effects, because if dependence severity and stress increase overall substance-related responding, this might limit the ability to detect a PIT effect, as there is less room for further augmentation in response to the reward-related stimulus. Thus, future studies may use an adapted version of a PIT paradigm as for example suggested by Seabrooke and colleagues (Seabrooke et al. 2017). In line with this, it can be hypothesised that different results with regard to the ‘chocolate PIT’ effect might have been observed in participants with addiction like sweet eating and loss of control over chocolate consumption.

In addition, due to limited personal resources, female participants were not observed in the SECPT by an experimenter of the opposite sex as previously suggested. As research demonstrated that the social evaluative component of the SECPT increases the cortisol response markedly (Schwabe et al. 2008), this might explain why in the present study, higher cortisol responses were observed for male compared with female participants, and the mean cortisol response was in general slightly lower compared with previous studies (Schwabe and Wolf 2009; Schwabe et al. 2008). In addition, lower cortisol responses in female compared with male participants may also be due to the effects of the menstrual cycle on the cortisol response. Thus, for example Maki et al. (2015) reported a significant increase in cortisol after a stress induction only in female participants tested in the follicular but not the luteal phase of the menstrual cycle. In the present study, testing for female participants was scheduled in the luteal phase which might also explain lower cortisol levels. However, our results were backed up by post hoc analysis including cortisol responders only, which supported our main findings.

Finally, compared with previous studies (Vogel et al. 2018), the number of participants that were classified as aware of the experimental contingencies was slightly lower. Although the number of participants in each group was still comparable with our previous experimental studies (Loeber and Duka 2009), replication of our findings in a larger sample of participants is warranted to exclude that the non-significant three-way interaction is due to lacking power. Related to this, we did not assess awareness of the experimental contingencies after the stress induction and can therefore not exclude that stress affected consolidation and/or retrieval of the experimental contingencies as previous studies demonstrated for example that stress affects memory retrieval (e.g. Maki et al. 2015). However, given that a ‘smoking PIT’ as well as a ‘chocolate PIT’ effect was observed in stressed participants, it is unlikely that lacking effects of the stress induction are confounded by impairing effects of stress on awareness of the experimental contingencies. Nevertheless, this aspect should be taken into account in future studies investigating the impact of stress on PIT effects.

To conclude, the present findings extend the results from Pool and colleagues (Pool et al. 2015) and Pritchard and colleagues (Pritchard et al. 2018) by suggesting that acute stress does affect the impact neither of a smoking-related stimulus on instrumental responding for a smoking-related reward nor of a chocolate-related stimulus for a chocolate-related reward. Thus, future studies are highly necessary to enhance our understanding of the interplay of stress and reward-related responding and mechanisms underlying the maintenance of addictive behaviour.
